# The Histone Chaperones SET/TAF‐1β and NPM1 Exhibit Conserved Functionality in Nucleosome Remodeling and Histone Eviction in a Cytochrome *c*‐Dependent Manner

**DOI:** 10.1002/advs.202301859

**Published:** 2023-08-07

**Authors:** Pedro Buzón, Alejandro Velázquez‐Cruz, Laura Corrales‐Guerrero, Antonio Díaz‐Quintana, Irene Díaz‐Moreno, Wouter H. Roos

**Affiliations:** ^1^ Moleculaire Biofysica Zernike Instituut Rijksuniversiteit Groningen Nijenborgh 4 Groningen 9747 AG The Netherlands; ^2^ Instituto de Investigaciones Químicas (IIQ) Centro de Investigaciones Científicas Isla de la Cartuja (cicCartuja) Universidad de Sevilla – Consejo Superior de Investigaciones Científicas (CSIC) Avda. Américo Vespucio 49 Sevilla 41092 Spain; ^3^ Present address: Department of Biochemistry University of Zurich Zurich 8057 Switzerland

**Keywords:** fluorescence microscopy, nucleophosmin 1, optical tweezers, SET/template‐activating factor‐Iβ, single‐molecule methods

## Abstract

Chromatin homeostasis mediates essential processes in eukaryotes, where histone chaperones have emerged as major regulatory factors during DNA replication, repair, and transcription. The dynamic nature of these processes, however, has severely impeded their characterization at the molecular level. Here, fluorescence optical tweezers are applied to follow histone chaperone dynamics in real time. The molecular action of SET/template‐activating factor‐Iβ and nucleophosmin 1—representing the two most common histone chaperone folds—are examined using both nucleosomes and isolated histones. It is shown that these chaperones present binding specificity for fully dismantled nucleosomes and are able to recognize and disrupt non‐native histone‐DNA interactions. Furthermore, the histone eviction process and its modulation by cytochrome *c* are scrutinized. This approach shows that despite the different structures of these chaperones, they present conserved modes of action mediating nucleosome remodeling.

## Graphical abstract

This study focuses on the molecular action of two histone chaperones: SET/template‐activating factor‐Iβ and nucleophosmin 1. It is shown how these chaperones have specificity for fully dismantled nucleosomes, characterized the histone eviction (removal) process, and its modulation by cytochrome *c*. Overall, it is shown that these chaperones exhibit conserved modes of action mediating nucleosome remodeling despite their structural differences.

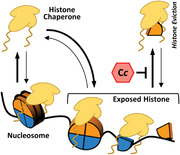

## 1 Introduction

The nucleosome is the minimal unit of structural organization in eukaryotic genomes.^[^ ^1^ ^]^ It comprises ≈146 base pairs (bp) of DNA that wraps around an octameric protein complex involving two copies of each core histone (H2A, H2B, H3, and H4).^[^ ^2^ ^]^ This supramolecular arrangement provides genome stability and confinement while serving as the ultimate regulatory barrier that mediates DNA accessibility. Thus, nucleosome dynamics plays a unique role in many of the most fundamental cellular processes—from genome replication and repair to gene expression. Nucleosome homeostasis is highly regulated and therefore coordinated by the action of many different nuclear factors including histone‐modifying enzymes,^[^ ^3^ ^]^ ATP‐dependent chromatin remodelers,^[^ ^4^ ^]^ and histone chaperones.^[^ ^5^ ^]^ In addition to the nuclear machinery, other cellular factors have been identified to play critical roles in diverse processes associated with chromatin remodeling.^[^ ^6^ ^]^

In this framework, histone chaperones have emerged as the main factors of the cellular machinery responsible for regulating histone availability.^[^ ^7^ ^]^ These chaperones are often multifunctional proteins involved in many processes related to histone metabolism such as folding, oligomerization, transport, deposition and eviction, storage, post‐translational modifications, and nucleosome assembly.^[^ ^5^, ^8^ ^]^ In general, histone chaperones perform their tasks by direct association with single and oligomeric histones, mediating specific interactions and preventing aggregation. Interestingly, histone chaperones also modulate antagonistic processes: histone‐DNA deposition and eviction. However, our understanding of the mechanisms behind these chaperoning processes is still limited. Moreover, histone chaperone activities are found in a wide range of different protein families, which show little sequence similarities between them. Nevertheless, two characteristic structural folds have been identified for their specific role as histone chaperones: the dimeric Nucleosome Assembly Protein 1‐like (NAP1‐like) fold and the pentameric nucleoplasmin fold.^[^ ^8^ ^]^ The structure‐function relationship of histone chaperones, for example, relating to the difference between dimeric and pentameric chaperones, remains however elusive.

The study of histone chaperone activities has proven experimentally challenging due to the intrinsic instability of histones and their aggregation propensities, particularly in the presence of DNA. Here, we present a set of single‐molecule manipulation strategies, combining optical tweezers (OT) and confocal fluorescence microscopy (CFM),^[^ ^9^ ^]^ to investigate chaperone activity in real‐time with molecular resolution. We selected two chaperones that represent the two most conserved histone‐chaperone folds: SET/template‐activating factor‐Iβ (SET/TAF‐Iβ) that presents the dimeric NAP1 fold,^[^ ^10^ ^]^ and nucleophosmin 1 (NPM), which exhibits the nucleoplasmin fold^[^ ^11^ ^]^ (Figure S1, Supporting Information). Specifically, we have probed the chaperone activity of both SET/TAF‐Iβ and NPM acting on individual histones, as well as in the context of the nucleosome. These chaperones showed binding specificity for fully disrupted nucleosomes, suggesting histone exposure as a key factor during nucleosome recognition. Moreover, the relative decrease in histone‐DNA affinity in the presence of chaperones was characterized through kinetic measurements, while providing direct observations on histone shielding and eviction. Finally, we studied the effect of the mitochondrial hemeprotein cytochrome *c* (C*c*). This protein has been recently reported to be translocated into the nucleus in the context of DNA damage, where it acts as a histone chaperone inhibitor.^[^ ^6^, ^12^ ^]^ Histone eviction assays in the presence of C*c* revealed that the disturbance of the chaperoning activity is coupled with the accumulation of chaperone molecules around DNA, suggesting new mechanisms for chaperone activity regulation. Hereby our results provide new molecular insights into the regulatory functions of histone chaperones coordinating histone‐DNA interactions.

## 2 Results

### 2.1 Both SET/TAF‐Iβ and NPM Exhibit Specificity for Unwrapped Nucleosomes

Histone chaperones are known to mediate various processes involved in the regulation of histone‐DNA interactions. One of their main functions is to assist nucleosome assembly and disassembly through histone deposition and eviction. To investigate these highly dynamic processes with molecular resolution, we employed a combination of OT and CFM. OT allows monitoring the mechanical unwrapping of reconstituted nucleosomes by performing force‐extension curves (FECs) on individual DNA molecules, while CFM provides direct visualization of fluorescently labeled chaperones and histones with single‐molecule resolution.

**Figure 1:**
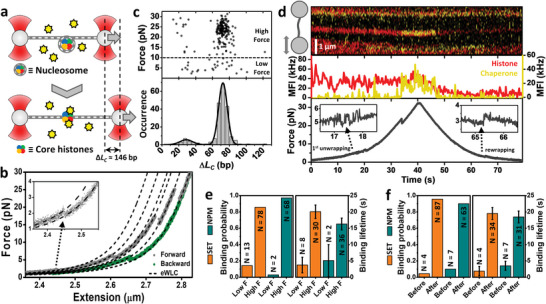
Chaperone‐histone interactions in the context of the nucleosome. a) Scheme of a nucleosome unwrapping experiment. Nucleosomes were mechanically unwrapped in the presence of 1–2 nm concentration of chaperone. Δ*L*_c_ shows the expected total change in contour length upon unwrapping a single nucleosome. b) Representative FEC showing several unwrapping events during the forward curve (dark grey) and rewrapping during the backward curve (green). Inset, zoom in on the first unwrapping event of the forward curve. The two high‐force (>10 pN) unwrapping events represent full nucleosome unwrapping. Black dashed lines represent eWLC fits to the forward curve (see Experimental Section). c) Lower panel, histogram of all Δ*L*_c_ values obtained from FECs of reconstituted nucleosomes (*N* = 208) under different conditions. The individual histograms are shown in Figure S2, Supporting Information. The fitting of the distribution to a double Gaussian reported 72 ± 5 and 29 ± 8 bp (center ± SD). Upper panel, scatter plot of the forces at which the changes in *L*_c_ occurred. d) Correlated FEC and fluorescence imaging showing histone eviction upon nucleosome unwrapping. Upper panel, kymograph recorded at 4 Hz of a fluorescently labeled nucleosome (red) and SET/TAF‐Iβ (yellow) during the FEC. Middle panel, mean fluorescence intensity (MFI) obtained from the traces shown in the upper panel, histones (red; left axis), and chaperone (yellow; right axis). Lower panel, force versus time plot of the FEC. Insets highlight the un/rewrapping events found at low forces. e) Left panel, binding probabilities obtained from FECs with SET/TAF‐Iβ (orange; *N* = 91 binding events) and NPM (dark green; *N* = 70) at low force (<10 pN) and high force (>10 pN). Right panel, the dwell time of the binding events identified below (low force) and above (high force) 10 pN, represented as mean ± SEM. *N* = 38 (SET/TAF‐Iβ) and *N* = 38 (NPM) binding events. The mean binding lifetimes obtained after unwrapping should be considered as lower limits of the real values, as some chaperones remained bound until the end of the experiment. f) Left panel, binding probabilities obtained for SET/TAF‐Iβ (orange; *N* = 91) and NPM (dark green; *N* = 70) before and after an unwrapping event was detected during the FEC. Right panel, dwell time of the binding events identified before and after any unwrapping event, represented as mean ± SEM. *N* = 38 (SET/TAF‐Iβ) and *N* = 38 (NPM) binding events. As stated in panel (e), the mean binding lifetimes obtained after unwrapping should be considered as lower limits of the real values.

Nucleosomes were in vitro reconstituted using ≈8.3 kbp DNA molecules lacking artificial nucleosome positioning sequences and isolated by using OT and microfluidics. Subsequently, individual molecules of DNA containing nucleosomes were brought to a solution of either SET/TAF‐Iβ or NPM, where nucleosomes were incubated for a minute before being mechanically unwrapped by pulling on the DNA (**Figure** **1**a). A representative FEC is shown in Figure 1b, displaying a typical saw‐tooth pattern during tether extension (grey) resulting from nucleosome unwrapping. Retraction curves (green) also showed abrupt changes in force, although to a lesser extent, indicative of DNA rewrapping. We use the extensible worm‐like chain (eWLC) model to fit the smooth regions of the FEC (dashed lines in Figure 1b) and determine the different values of apparent contour length (*L*_c_) of the tether (see Experimental Section). Then, from the values of *L*_c_, the changes in apparent contour length (Δ*L*_c_) after every rupture event of the curve are calculated, corresponding to the number of unwrapped base pairs (Figure 1b,c).

Our nucleosome unwrapping experiments reported a major Δ*L*_c_ population at 72 ± 5 bp (center ± SD) (Figure 1c), in agreement with the values reported in the literature.^[^ ^13^ ^]^ In addition, a smaller and wider peak was found at ≈30 bp (Figure 1c). This small population could be due to partial nucleosome unwrapping/rewrapping multistep events, smaller than the canonical ≈72 bp. Also, changes in nucleosome reorientation with respect to the direction of the applied force could be taking place, as previously characterized.^[^ ^13e,i^ ^]^ A recent nucleosome unwrapping study has revealed that only ≈13% of nucleosomes show cooperative unwrapping at low force (4 ± 5 pN; mean ± SD) under similar ionic strength conditions probed here, that is, 50 mm K^+^.^[^ ^13i^ ^]^ Although the resolution of our FECs did not allow to identify non‐cooperative unwrapping events, we found that ≈15% of unwrapping events at high force (>10 pN) show a cooperative unwrapping event below 10 pN (Table S1, Supporting Information), which allows us to rule out the presence of a substantial number of tetrasomes and hexasomes in our FECs. Overall, our experiments indicate that unwrapping is not influenced by the presence of chaperones, as neither the measured Δ*L*_c_ nor the force unwrapping patterns are changed (Figure S2a–d and Table S1, Supporting Information).

In addition to the *L*_c_ changes, fluorescently labeled histones (red, Figure 1d) and chaperones (yellow) were directly visualized by two‐color CFM. Nucleosome labeling did not report any differences in Δ*L*_c_ when compared to FECs of non‐labeled nucleosomes (Figure S2e,f, Supporting Information). We spotted two major actions when looking at the histone fluorescence signal in combination with force‐extension experiments. Histones were found to either remain bound to DNA or unbind after nucleosome unwrapping (Figure S3a,b, Supporting Information). Moreover, fluorescence experiments also revealed that in the absence of histones, using bare DNA molecules, chaperones do not interact with DNA, as discussed in the next sections.

By correlating the FECs with fluorescence imaging, we were able to reveal, in real‐time, the action of chaperones during histone eviction from partially unwrapped nucleosomes. Figure 1d shows how the SET/TAF‐Iβ signal (yellow) colocalizes with the histone signal (red) for ≈15 s followed by a correlated decrease in both signal intensities. Not all fluorescently labeled histones within the nucleosome are evicted by the chaperone, as perceived by the remaining red fluorescence intensity (Figure 1d). Eviction is identified by the time‐correlated decrease in the histone signal (red) and the drop of the chaperone signal (yellow) to background levels. According to our brightness calibration (Figure S4, Supporting Information), a fluorescently labeled SET/TAF‐Iβ protein presents ≈20 kHz (photons•10^3^/s) in mean fluorescence intensity (MFI). The dimeric nature of SET/TAF‐Iβ implies that on average every dimer displays two fluorophores (see Experimental Section), allowing us to identify a drop of ≈20 kHz as chaperone unbinding, as two fluorophores typically do not bleach simultaneously. Hence, Figure 1d shows an example capturing histone eviction carried out by an individual chaperone. Another example of eviction where the histone signal dropped to background levels is shown in Figure S3c, Supporting Information. Moreover, we also find examples where chaperones could either remain bound until the end of the FEC or unbind without any change in the histone signal (Figure S3d, Supporting Information). The latter observation might be conditioned by our nucleosome labeling procedure, which is based on the stochastic labeling of native lysine residues (see Experimental Section). Nevertheless, the obtained binding probabilities and binding lifetimes revealed that both chaperones have a clear preference for binding to nucleosomes at forces higher than 10 pN (Figure 1e). Specifically, more than 90% of the chaperone binding events were found after complete unwrapping at high force transitions, suggesting that histone exposure greatly favors the recognition of nucleosomes by these histone chaperones, which is further supported by the analysis of binding lifetimes (Figure 1f). The binding events identified after complete nucleosome unwrapping were significantly longer, indicative of the higher affinity of chaperones for fully exposed histones.

### 2.2 Histone Chaperones are Recruited to DNA by DNA‐Bound Histones

**Figure 2:**
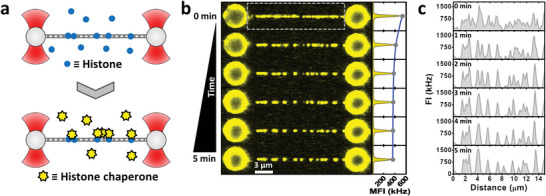
Direct visualization of histone recognition by chaperones. a) DNA molecules are stretched to a fixed distance, corresponding to 10 pN tension, and then incubated in a solution of individual core histones at 100 nm concentration for ≈30 s. Next, histone‐DNA complexes were brought to a solution of fluorescently labeled histone chaperones at 5 nm concentration, and observed for 5 min. b) Left panel, fluorescence images recorded at a one‐image‐per‐minute frequency (image time ≈5 s) during the incubation of an H2B‐DNA complex in the presence of 5 nm NPM. Right panel, MFI measured from the area between the beads (dashed rectangle) at each time point. c) Fluorescence intensity (FI) values corresponding to the signal of a single line scan of one‐pixel thickness along the DNA molecule shown in (b) at different time points.

To gain a better understanding of the mechanisms underlying the various observations described above, including chaperone‐histone interaction and eviction, we continued studying the chaperone activity of SET/TAF‐Iβ and NPM with the different histones (Figure S5, Supporting Information). DNA molecules (≈48 kbp; see Experimental Section) were isolated using OT and microfluidics, and incubated in a solution containing a single type of core histone (H2A, H2B, H3, or H4) to allow the formation of histone‐DNA complexes. Subsequently, these complexes were brought to a solution of fluorescently labeled histone chaperones to monitor their interaction (**Figure** **2**a). While control experiments without histones do not report any chaperone binding event, measurements including histones confirm that chaperones are recruited to DNA by DNA‐bound histones. Figure 2b shows the presence of chaperone‐histone‐DNA complexes and their evolution during the first 5 min of incubation in the chaperone solution. CFM imaging reveals how the fluorescence signal at certain regions of the DNA molecule disappears during the incubation time, indicative of chaperone unbinding (Figure 2b,c). In addition, the overall signal measured as MFI reported that the major changes in fluorescence occur during the first 2–3 min (Figure 2b, right panel). All core histones behaved similarly and showed the same trend; for both SET/TAF‐Iβ and NPM the fluorescence signal disappeared partially, but not completely, within 5 min (Figure S6a, Supporting Information).

By continuously scanning along the DNA molecule at a much higher frequency, we were able to capture the truly dynamic nature of the process (Figure S6b, Supporting Information). We observed a fast binding of chaperones, followed by a clear decrease in fluorescence intensity within 3 min. As chaperones do not bind to bare DNA under our conditions, these results suggest that the number of DNA‐bound histones decreases over time upon incubation with histone chaperones, supporting our observations of histone eviction in the context of mechanically unwrapped nucleosomes.

### 2.3 Histone Chaperones Prevent DNA Bridging through Histone Shielding and Eviction

**Figure 3:**
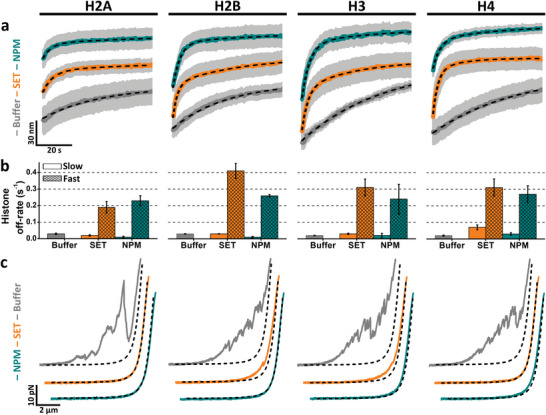
Eviction and shielding of DNA‐bound histones mediated by histone chaperones. a) Averaged kinetic traces of relative bead‐to‐bead distances measured over time for histone‐DNA complexes during their incubation against buffer (grey), 5 nm SET/TAF‐Iβ (orange), and 5 nm NPM (dark green). Averaged curves were generated from individual traces (*N* ≥ 5) and fitted to either a single or double exponential function (black dashed line) for the first 2 min of incubation. For visualization purposes, only the first minute is shown. Grey shades represent SEM. b) Off‐rates obtained from the fits presented in (a). Traces obtained in the buffer could be fitted to a single exponential, while chaperone traces presented two off‐rates. Error bars represent SEM. c) Representative FECs obtained after 5 min incubation in buffer (grey), 5 nm SET/TAF‐Iβ (orange), and 5 nm NPM (dark green). Black dashed lines represent simulated bare DNA curves generated with the eWLC model and experimentally measured parameters (see Experimental Section).

To further study histone‐DNA unbinding kinetics, we applied a strategy based on measurements of DNA de/condensation upon protein un/binding. This assay, which is independent of fluorescence intensity measurements, allows monitoring histone binding with ten times higher temporal resolution, that is, ≈100 Hz, instead of the ≈10 Hz obtained by confocal scanning (Figure S6b, Supporting Information). DNA molecules held in the presence of histones experienced an increase in tether tension upon binding, and hence, a decrease in the bead‐to‐bead distance (Figure S7a, Supporting Information). Both tether tension and bead‐to‐bead distance get partially restored to their original, that of bare DNA, when histone‐DNA complexes are brought into the chaperone solution (**Figure** **3**a and Figure S7a, Supporting Information). Figure 3a shows averaged kinetic traces of bead‐to‐bead distances for all individual histone‐DNA complexes upon incubation with either NPM (dark green), SET/TAF‐Iβ (orange), or buffer (grey) as control.

Histone‐DNA complexes that were brought to the buffer solution showed a monotonic increase in bead‐to‐bead distance reporting similar off‐rates, between 0.02 and 0.03 s^−1^ (Figure 3a,b). However, in the presence of chaperones, significantly faster kinetics were found in all cases. These traces showed a bimodal growth composed of a slow component, which resembles the off‐rates measured in the buffer, and a second kinetic component approximately ten times faster (Figure 3a,b). These results are in agreement with the behavior revealed by CFM experiments, in which the amount of bound chaperones decreased over time (Figure 2b,c). In addition, this kinetic analysis indicates that histones are being actively removed, evicted, from DNA by the action of histone chaperones. To further validate this, we used a truncated version of SET/TAF‐Iβ (SET/TAF‐Iβ‐ΔC), lacking the C‐terminal disordered acidic domain and displaying a lower affinity for histones.^[^ ^10^ ^]^ As could be anticipated, kinetic traces in the presence of SET/TAF‐Iβ‐ΔC showed a clear decrease in the histone eviction rate when compared with full‐length SET/TAF‐Iβ (Figure S7b,c, Supporting Information).

We then attempted to gain further insights into the chaperone‐histone complexes that remained bound to DNA (Figure 2b,c and Figure S6, Supporting Information). Thus, FECs of histone‐bound DNA were performed after 5 min incubation in either buffer or chaperone solution. FECs without chaperone displayed a large amount of DNA loops, generated during the relaxation of the tether tension, and disrupted during pulling (Figure 3c). These DNA loops are most likely formed through histone‐DNA interactions between distant regions of the DNA molecule, and nonspecific histone–histone interactions. Interestingly, when FECs were performed in the presence of either of the chaperones, the curves turned substantially smoother, in some cases resembling bare DNA curves (Figure 3c). These experiments, combined with CFM imaging (Figure 2b and Figure S6, Supporting Information), show the capabilities of both SET/TAF‐Iβ and NPM to accumulate on DNA‐bound histones and shield nonspecific histone–histone and histone‐DNA interactions, preventing DNA bridging.

Ultimately, our results unveil, at the molecular level, two major regulatory functions of chaperones in the context of chromatin remodeling. Both SET/TAF‐Iβ and NPM showed the ability to efficiently disrupt noncanonical histone‐DNA interactions through histone removal. When histone eviction cannot be achieved, histone shielding is used to prevent the formation of further nonspecific interactions, thus ensuring genome integrity.

### 2.4 Cytochrome *c* Impairs the Chaperone Activity of SET/TAF‐Iβ and NPM

C*c* is a multifunctional^[^ ^14^ ^]^ mitochondrial protein known to play a major role in the electron transport chain,^[^ ^15^ ^]^ a key process in the synthesis of ATP. In addition, C*c* has been shown to be released from mitochondria and translocate into the nucleus under DNA damage conditions.^[^ ^6^, ^16^ ^]^ Although the nuclear role of C*c* is not fully understood, it has recently been shown that following DNA breaks, C*c* specifically interacts with SET/TAF‐Iβ,^[^ ^6a^ ^]^ and NAP1‐Related Protein 1 (NRP1),^[^ ^6b^ ^]^ a plant homologous of SET/TAF‐Iβ, acting as a histone chaperone inhibitor. Therefore, we decided to include C*c* in our single‐molecule kinetic experiments to challenge the relevance of the chaperone activities reported in Figure 3.

**Figure 4:**
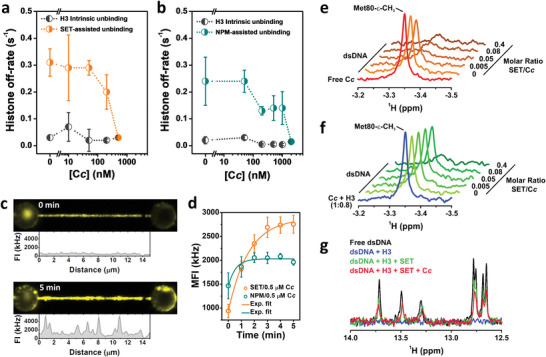
C*c* represses histone eviction by inhibiting chaperone activity. a) Off‐rates of H3 dissociation from DNA as a function of C*c* concentration (10–500 nm) and in the presence of 5 nm SET/TAF‐Iβ, as obtained from averaged kinetic traces. The slow (H3 intrinsic unbinding) rate is depicted in black, while the fast (SET/TAF‐Iβ assisted unbinding) rate is colored in orange. Error bars represent SEM. b) Off‐rates obtained as described in (a) for a solution comprising 5 nm NPM and varying concentrations of C*c* (50 nm–2 µm). NPM‐assisted unbinding is colored in dark green and the intrinsic H3 unbinding rate in black. Error bars represent SEM. c) Representative fluorescence images of an H3‐DNA complex in a solution of 5 nm SET/TAF‐Iβ and 500 nm C*c* at 0 and 5 min incubation. FI values correspond to the signal of an individual line scan along the DNA molecule. d) MFI over time obtained from CFM images as the fluorescence images shown in (c), for SET/TAF‐Iβ (orange; *N* = 5) and NPM (dark green; *N* = 3), both with 500 nm of C*c*. Error bars represent SEM. Fits to an exponential function (solid lines) reported 0.64 ± 0.07 s^−1^ (SET/TAF‐Iβ) and 2 ± 1 s^−1^ (NPM). e) 1D ^1^H NMR spectra monitoring the Met80‐ε‐CH_3_ signal of reduced C*c*, mixed with dsDNA oligo in a fixed ratio (1:2) and increasing concentrations of SET/TAF‐Iβ. f) The same experiment as before but including a fixed amount of H3 at each point of the titration. g) Superimposition of the base‐pairing hydrogen bond signals from 1D ^1^H NMR spectra of dsDNA oligo, either alone (black) or upon sequential addition of H3 (blue), SET/TAF‐Iβ (green) and C*c* (red) at a molar ratio of 1:0.4:0.025:0.5 (dsDNA:H3:SET/TAF‐Iβ:C*c*).

Due to the very similar behavior exhibited by all core histones (Figure 3 and Figure S6, Supporting Information), kinetic experiments were performed only with histone H3, measuring SET/TAF‐Iβ and NPM chaperone activity at increasing concentrations of C*c* (**Figure** **4**a,b). Explicitly, H3‐DNA complexes were brought to a mixture solution of chaperone and C*c*, keeping chaperone concentration constant (5 nm), while varying C*c* concentration. We found a clear decrease in the fast kinetic component with increasing C*c* concentrations, proving that histone eviction activity is weakened by C*c* (Figure 4a,b). Particularly, chaperone activity was completely inhibited at 0.5 (for SET/TAF‐Iβ) and 2 µm (for NPM) concentration of C*c*. The same behavior was observed with SET/TAF‐Iβ‐ΔC (Figure S7d, Supporting Information). Control experiments performed with C*c* at these saturating concentrations, but without chaperones, did not alter the H3 intrinsic unbinding rate (Figure S8a, Supporting Information). The inhibition of histone shielding could not be addressed as C*c* interacts with DNA and promotes the formation of DNA loops by itself, even in the presence of histone chaperones (Figures S8b–d and S9a,b, Supporting Information). Similarly, nucleosome unwrapping could not be detected in the presence of C*c* as the disruption of these DNA loops showed much greater signals than the ones obtained from nucleosome disruption.

In addition to the kinetic analysis, CFM imaging of fluorescently labeled chaperones in the presence of C*c* also pointed toward a reduction in histone removal activity. Figure 4c shows how C*c* promotes chaperone accumulation onto DNA upon incubation, reverting the behavior described previously for chaperones in the absence of C*c*, where fluorescence intensity would decrease over time (compare with Figure 2b,c). Chaperone‐C*c* localization on DNA showed a monotonic increase over the first minutes of incubation before reaching saturation (Figure 4d). We report chaperone accumulation rates of 0.64 ± 0.07 (SET/TAF‐Iβ) and 2 ± 1 s^−1^ (NPM), both at 500 nm of C*c*. Consequently, chaperones appear to be still able to interact with histones even when C*c* is present, while histone eviction activity is inhibited. Control experiments carried out without histones showed that C*c* has also the ability to bind to DNA and recruit chaperones (Figure S8e–h, Supporting Information), although to a much lesser extent than in the presence of histones (Figure 2b,c).

To gain a better insight into the molecular mechanism behind the disruptive effect of C*c* on the histone chaperone function, a series of complementary NMR measurements were carried out. Competition between C*c* and histones for binding to several chaperones, including SET/TAF‐Iβ, was previously reported.^[^ ^6^, ^12^, ^17^ ^]^ However, none of these assays were performed in the presence of DNA, an essential element in the physiological context of the interactions examined. For that reason, 1D ^1^H NMR experiments with samples containing a 10‐mer double‐stranded DNA (dsDNA) were conducted following the Met80 methyl proton signal (ε‐CH_3_) of reduced C*c* and the base‐pairing hydrogen bond signals from the dsDNA. A mixture of C*c* and dsDNA was titrated with increasing concentrations of SET/TAF‐Iβ, either in the presence or absence of histone H3. C*c* signal intensity (Met80) decreased upon the addition of dsDNA and increasing concentrations of the chaperone as a consequence of the longer diffusional correlation time of the hemeprotein in the complex, leading to faster transverse relaxation (Figure 4e). This effect was hampered by H3 due to direct competition for both dsDNA and SET/TAF‐Iβ (Figure 4f and Figure S9b, Supporting Information). Furthermore, C*c* could also competitively attenuate SET/TAF‐Iβ‐mediated histone eviction, as indicated by the dsDNA signals (Figure 4g), in line with OT and CFM observations (Figure 4a–d). Finally, the direct association of SET/TAF‐Iβ with dsDNA (Figure S9c, Supporting Information) and C*c* with histone H3 (Figure S9d, Supporting Information) was discarded.

Taken together, these results reveal what seems to be a very specific function of C*c* during chaperone activity regulation, which involves the inhibition of histone eviction while promoting chaperone accumulation around the DNA; a function that could potentially be conserved for many other histone chaperones.

## 3 Discussion

Correlated OT and single‐molecule fluorescence measurements revealed that both SET/TAF‐Iβ and NPM are recruited to DNA by histones. Previous quantification of the binding affinity of SET/TAF‐Iβ and NPM for histones in the absence of DNA provided dissociation constants (*K*_D_) in the micromolar concentration range by isothermal titration calorimetry.^[^ ^6a^ ^]^ Although these histone chaperones have also been described to exhibit significant DNA binding capabilities,^[^ ^10^, ^18^ ^]^ we have shown that in the low nanomolar range, they specifically colocalize onto DNA only through other DNA‐binding proteins. Transient binding of chaperones to DNA might still occur, but our experiments show that those must be in the order of ≈200 ms or shorter, according to the temporal resolution achieved by fluorescence microscopy. Our kinetic analysis of histone‐DNA interactions reported off‐rates between 0.02 and 0.03 s^−1^. These values represent the kinetic constant (*k*_off_) for individual histones under moderate ionic strength conditions (50 mm KCl). Assuming a *k*_on_ between 10^8^–10^6^ M^−1^s^−1^, our results suggest a high affinity between histones and DNA, with *K*_D_ values between the picomolar and low nanomolar range. Affinities that are consistent with estimations of the DNA binding properties of histone octamers.^[^ ^19^ ^]^ Importantly, these affinities decrease approximately ten times when 5 nm of chaperone is added to the solution (Figure 3a,b), proving SET/TAF‐Iβ and NPM histone eviction mechanisms. Histone eviction activity was further visualized by CFM, monitoring the decrease of bound chaperones over time. However, core histones are known to arrange as heterodimers, H2A‐H2B and H3‐H4, or tetramers in the case of (H3‐H4)_2_, prior to their deposition on DNA.^[^ ^20^ ^]^ Therefore, our observations quantitatively describe the decisive ability of these chaperones to recognize non‐canonical histone‐DNA interactions, where histones are not in their dimeric arrangement, and trigger histone eviction. These findings are in line with the reported NAP1 activity mediating the interaction between H2A‐H2B dimers and DNA.^[^ ^21^ ^]^

Fluorescence imaging of chaperone‐histone‐DNA complexes also showed that not all histones are removed during chaperone incubation. These complexes were always found to be distributed as discrete high‐intensity spots (Figure 2b and Figure S6, Supporting Information), instead of a homogeneous low‐intensity coverage of DNA molecules. Furthermore, our estimation of the count rate per fluorophore (Figure S4, Supporting Information) suggests that these complexes include tens, and sometimes hundreds, of chaperone molecules per diffraction‐limited spot. Hence, we hypothesize that the remaining chaperone‐histone complexes are composed of histone aggregates that cannot be removed by the action of the chaperones. Nonetheless, both SET/TAF‐Iβ and NPM displayed an additional mechanism to tackle these aggregates and prevent non‐canonical histone‐DNA interactions. FECs (Figure 3c) in combination with CFM imaging (Figure 2b and Figure S6, Supporting Information) showed that these chaperones can gather around histone aggregates and efficiently shield their DNA interacting regions. Thus, chaperones avoid the formation of nonspecific DNA‐histone interactions, and hence DNA bridging, which explains their capabilities to prevent genome condensation and preserve its integrity. We hypothesize that during nucleosome remodeling, monomeric histones could be present in the vicinity of the genome and interact with it. Therefore, both eviction and shielding could be essential chaperone functions to ensure the execution of downstream processes.

**Figure 5:**
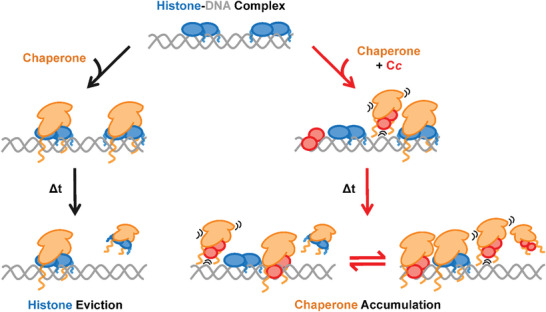
Schematic representation of C*c*‐mediated dysregulation of histone chaperone activity. Chaperones (orange) bind to DNA‐associated individual core histones (blue) causing their eviction and subsequent shielding. However, when C*c* (red) is present, competition for chaperone binding arises between histones and the hemeprotein. Furthermore, C*c* is also capable of interacting with the DNA, creating on its surface additional binding sites for chaperones, although more transient (motion lines) than those of histones. This situation promotes a long‐lasting presence of chaperones onto DNA and its vicinity, permanently attracted by their DNA‐binding protein partners. In addition, DNA‐bridging events produced by histones and C*c* could contribute to the permanence of chaperones in the vicinity of the DNA.

C*c* has been shown to act as a histone chaperone inhibitor of NAP1‐like chaperones following DNA damage.^[^ ^6^ ^]^ Specifically, C*c* showed the capability to inhibit nucleosome assembly. Furthermore, C*c* was identified to compete with histones for binding to those chaperones, a phenomenon that we have now confirmed also occurs in the presence of DNA (Figure 4e,f), suggesting the capacity of the hemeprotein to displace chaperone‐histone complexes. Indeed, we have demonstrated that C*c* is able to repress histone eviction in a concentration‐dependent manner, but without fully blocking chaperone‐histone interactions (Figure 4a,b,g). Fluorescence imaging showed the accumulation of chaperones on the DNA when histones and C*c* are present (Figure 4c,d). In this line, while both NPM and SET/TAF‐Iβ were able to shield non‐canonical DNA interactions of histones in the absence of C*c* (Figure 3c), the addition of both DNA‐interacting proteins produced a combined DNA‐bridging effect (Figure S8b–d, Supporting Information), pointing to a very complex scenario (**Figure** **5**). Taken together, these observations provide new details on the mechanism of histone chaperone impairment mediated by C*c*, where the role of DNA would be indispensable.

Histone eviction activity by chaperones was not only shown to occur for individual histones but also for nucleosome‐forming core histones (Figure 1d). However, our nucleosome unwrapping experiments described a far more complex behavior than the one observed with individual histones. Eviction was not always detectable, as chaperones showed the capability to either remain bound for periods of time that would exceed the length of the experiment, or unbind without any change in the histone signal. In addition, our histone labeling strategy prevented us from performing a quantitative analysis of this chaperone activity, nor addressing questions related to chaperone specificity for H2A‐H2B or H3‐H4 dimers. Despite this, correlated fluorescence imaging and mechanical unwrapping experiments revealed that these chaperones do not affect nucleosome stability (Figure S2 and Table S1, Supporting Information), while showing a clear preference to interact with dismantled, or fully unwrapped, nucleosomes (Figure 1e,f). Specifically, these results disclose the importance of the exposure of certain core histone regions, otherwise buried in the context of the nucleosome, to the interaction with SET/TAF‐Iβ or NPM. These findings imply that neither SET/TAF‐Iβ nor NPM would have the ability to recognize or destabilize nucleosomes under our experimental conditions. Other chaperones have proven their capacity to destabilize nucleosomes in vitro: NAP1,^[^ ^22^ ^]^ nucleolin,^[^ ^23^ ^]^ and FAcilitates chromatin transcription (FACT).^[^ ^24^ ^]^ With the exception of NAP1, which shares structure with SET/TAF‐Iβ, FACT and nucleolin do not present structural or sequence similarities to SET/TAF‐Iβ or NPM.

Regarding the chaperones studied here, SET/TAF‐Iβ is known to mediate chromatin decondensation^[^ ^25^ ^]^ through its interaction with the linker histone H1,^[^ ^26^ ^]^ the chromatin remodeling protein prothymosin α,^[^ ^27^ ^]^ and the transcription coactivator cAMP‐response element binding (CREB)‐binding protein.^[^ ^28^ ^]^ Moreover, NPM has also been identified to directly interact with histone H1,^[^ ^29^ ^]^ promote acetylation‐dependent chromatin transcription,^[^ ^30^ ^]^ and mediate both nucleosome formation and chromatin decondensation,^[^ ^31^ ^]^ among other cellular functions.^[^ ^32^ ^]^ However, under the experimental conditions assayed here, these chaperones showed specificity for unwrapped nucleosomes and did not show any long‐lived interactions with intact nucleosomes. Thus, we hypothesize the following scenarios regarding SET/TAF‐Iβ and NPM functions: the recognition of intact nucleosomes is i) mediated by posttranslational modifications (PTMs) of chaperones/histones, not present in our assays; ii) or it occurs through the cooperation of these chaperones with other cellular factors; iii) or by a combination of the former and the latter. Overall, our data support the preferential binding of SET/TAF‐Iβ and NPM to dismantled nucleosomes in the absence of the abovementioned regulatory processes.

## 4 Conclusion

This work provided a mechanistically detailed view in real‐time of the actions of SET/TAF‐Iβ and NPM, representing two different families of histone chaperones. In summary, we have revealed a set of chaperone activities that seem to be conserved in the two most representative chaperone folds found in eukaryotes, the NAP1‐like and nucleoplasmin‐like folds. Both SET/TAF‐Iβ and NPM exhibited specificity for fully unwrapped nucleosomes, as well as for DNA‐bound histones in the absence of nucleosomes. These chaperones also showed the ability to efficiently frustrate non‐nucleosomal interactions between histones and DNA, promoting histone eviction. Moreover, the uncovered features of the inhibitory mechanism exerted by C*c* indicated that chaperone activities can be suppressed without chaperone‐histone interactions being fully hindered. Thus, our results evince that these histone chaperones share fundamental activities, mediating histone recognition in a very similar manner—potentially present in many other histone chaperones. Hereby, we have provided new molecular details into the mechanisms behind these processes mediated by histone chaperones as well as their regulation.

## 5 Experimental Section

### Protein Samples

The pET3a expression plasmids coding for core histones from *Xenopus laevis* were obtained from Dr. Tim Richmond (Institute of Molecular Biology and Biophysics, Switzerland). *X. laevis* histones share ≥94% sequence identity with human histones and were shown to interact with both SET/TAF‐Iβ^[^ ^10^ ^]^ and NPM.^[^ ^29^ ^]^ Human NPM and SET/TAF‐Iβ wild type constructs were cloned in a frame with an N‐terminal 6xHis‐tag in pET28a(+) vectors. The DNA coding for human C*c* was in a pBTR1 plasmid,^[^ ^33^ ^]^ along with the yeast heme lyase for proper protein folding.

For the fluorescent labeling of SET/TAF‐Iβ and C*c* by maleimide derivatization, a cysteine was introduced in their sequences by site‐directed mutagenesis. The point mutation Q69C was inserted into the full‐length (SET/TAF‐Iβ‐Q69C) and the C‐terminal deletion (SET/TAF‐Iβ‐ΔC‐Q69C, amino acids 1–225) constructs as reported.^[^ ^34^ ^]^ The C*c*‐E104C mutant was produced as previously described.^[^ ^35^ ^]^

All proteins were expressed in *Escherichia coli* BL21(DE3) electrocompetent cells and grown in Luria–Bertani medium. Core histones were purified according to the protocol reported by Luger et al.^[^ ^36^ ^]^ with minor modifications. Explicitly, upon expression of each *X. laevis* core histone, cells were harvested at 5000 g and 4 °C for 10 min. Cell pellets were resuspended in lysis buffer (50 mm Tris‐HCl, 100 mm NaCl, and 1 mm β‐mercaptoethanol [βME], pH 7.5), sonicated (Branson Ultrasonics Sonifier SFX550 Cell Disruptors; 30 s ON, 30 s OFF, for 2 min at 40% amplitude, on ice) and centrifuged at 28 000 × *g* and 4 °C for 10 min. The supernatant was discarded and broken cell pellets were first washed three times with lysis buffer supplemented with 1% Triton X‐100, then washed twice with just lysis buffer (without detergent), and finally soaked with 1 mL dimethyl sulfoxide (DMSO) by stirring with a spatula and vortexing for 15–20 min to solubilize inclusion bodies, where histones were accumulated. Next, extracted histones were denatured by adding 49 mL of unfolding buffer (50 mm Tris‐HCl buffer, 6 m guanidinium chloride, and 1 mm dithiothreitol [DTT], pH 7.5) and applying constant shaking until complete homogenization and dissolution of visible particles (for ≈2–3 h). The mixture was then centrifuged at 28 000 × *g* and 25 °C for 10 min, and the supernatant was dialyzed three times, with changes every ≈12 h, in 5 L of Milli‐Q water with 5 mm βME. After dialysis, the histone solution was centrifuged at 3000 g and 25 °C for 10 min; and the precipitate was isolated and resuspended in SAU buffer (20 mm sodium acetate, 7 m urea, 2 mm disodium ethylenediaminetetraacetate [EDTA‐Na_2_], and 1 mm βME, pH 5.2). Upon complete dissolution of the precipitate by gently stirring with a spatula, it was centrifuged again at 28 000 × *g* and 25 °C for 10 min, and the supernatant containing solubilized histones was recovered. Subsequently, purification was carried out by gravity‐flow cation exchange chromatography with a carboxymethylcellulose column (Whatman). A non‐continuous gradient of NaCl between 0 and 0.5 m in SAU buffer was applied and then elution fractions were analyzed by sodium dodecyl sulfate‐polyacrylamide gel electrophoresis (SDS‐PAGE). Fractions containing pure histones were dialyzed three times, with changes every ≈12 h, in 5 L of Milli‐Q water. Later, samples were flash‐frozen with liquid N2 and lyophilized. Finally, histones were resuspended in Milli‐Q water and centrifuged at 28 000 × *g* and 4 °C for 10 min, to eliminate any precipitate. The resulting supernatant contained pure and folded histones, as shown by SDS‐PAGE and circular dichroism (CD) (Figure S5, Supporting Information).

C*c* purification, either wild‐type (WT) or E104C, was performed by cation exchange chromatography, with a Nuvia S column (Bio‐Rad) and using a fast protein liquid chromatography (FPLC) system (NGC Chromatography System Quest 10, Bio‐Rad).

The concentration of individual histones was estimated using the Bradford assay,^[^ ^37^ ^]^ whereas C*c* was quantified in its reduced state by measuring its absorbance at 550 nm (ε_550_ = 28.92 mm ^−1^ cm^−1^). All proteins were stored at −80 °C until use.

### Fluorescent Labeling of Histones Chaperones

Both chaperones SET/TAF‐Iβ‐Q69C and NPM were fluorescently labeled using Alexa Fluor 532 C_5_ maleimide (ThermoFisher). NPM was labeled by means of its endogenous exposed Cys104. Cys reduced state was ensured by incubating the chaperones at low micromolar concentrations with 10 mm DTT for 30 min on ice. DTT was removed by applying the mixture to a PD Minitrap G‐25 column (GE Healthcare). Labeling reactions were carried out by adding 1:10 excess of fluorophore reagent to Cys residue and incubating for 2 h at 4 °C. After incubation, the reaction was ended by adding 10 mm DTT, and the excess of dye was removed by using a PD Minitrap G‐25 column for NPM or a Superdex 200 10/300 GL (GE Healthcare) size exclusion chromatography column for SET/TAF‐Iβ. Final protein concentration and labeling efficiency were determined spectrophotometrically according to the guidelines provided by the manufacturer (ThermoFisher). Labeling efficiencies were ≈95% and ≈75% for SET/TAF‐Iβ and NPM, respectively, implying two fluorophores per dimeric SET/TAF‐Iβ molecule and four fluorophores per NPM pentamer on average. Chaperone concentrations were determined spectrophotometrically at 280 nm (ε_280_ = 16 960 M^−1^ cm^−1^ for NPM and ε_280_ = 32 430 M^−1^ cm^−1^ for SET/TAF‐Iβ and SET/TAF‐Iβ‐ΔC). NPM and SET/TAF‐Iβ(−Δ*C*) concentrations were expressed in their pentameric and dimeric forms, respectively. Labeled proteins were flash‐frozen and stored at −80 °C.

### Nucleosome Assembly and Fluorescent Labeling

Nucleosome reconstitution was performed by salt dialysis on biotinylated DNA (pKYB1 vector; New England Biolabs) without any nucleosome positioning sequence, similar to previously reported protocols.^[^ ^38^ ^]^ An equimolar mixture (75 nm) of *X. laevis* core histones in a volume of 50 µL was incubated on ice for 30 min in high salt buffer (25 mm 4‐(2‐hydroxyethyl)−1‐piperazineethanesulfonic acid [HEPES], 2 m KCl, 1 mm ethylenediamine tetraacetic acid [EDTA], 10 mm DTT, 0.1 mg mL^−1^ bovine serum albumin [BSA], adjusted to pH 7.5). Afterward, 2 ng µL^−1^ of biotinylated DNA was added to the mixture to a final volume of 100 µL and further incubated on ice for 30 min. Then, the 100 µL mixture of DNA and core histones was put into a Slide‐A‐Lyzer device of 7000 MWCO (ThermoFisher) and dialyzed overnight against low salt buffer (25 mm HEPES, 50 mm KCl, 0.1 mm EDTA, pH 7.5). Reconstituted nucleosomes were diluted 20 times in low salt buffer before they were applied into the microfluidic chamber; otherwise, samples were stored at 4 °C for up to 3 days. Under these assembly conditions, DNA molecules were found that would typically show from 0 to 6 unwrapping events during force‐spectroscopy experiments. The number of unwrapping events detected in the high force regime (Table S1, Supporting Information) reported an average number of two nucleosomes per DNA molecule.

Fluorescent labeling was performed by targeting histone amine groups of pre‐assembled nucleosomes using Alexa Fluor 647 succinimidyl ester (ThermoFisher), following the previously reported strategy.^[^ ^13g^ ^]^ A mixture of ≈2 ng µL^−1^ DNA with reconstituted nucleosomes, and 200 µm fluorophore reagent, was set in 25 mm HEPES, 50 mm KCl, 0.1 mm EDTA, 0.1 mg mL^−1^ BSA, 0.02% (v/v) Tween 20, 2% DMSO, pH 7.5. After 1‐h incubation at room temperature, the reaction was diluted 20 times in low salt buffer and used in the microfluidic cell. Labeled nucleosomes were prepared fresh and discarded at the end of the day.

The suitability of the purified *X. laevis* core histones and the pKYB1 vector for in vitro nucleosome assembly was checked using an MNase digestion assay. The plasmid was purified by alkaline lysis as previously described^[^ ^6a^ ^]^ and then linearized by double restriction enzyme digestion with EcoRI and KpnI (New England Biolabs), according to the manufacturer's instructions. The complete linearization of the plasmid was confirmed by electrophoresis on 1% agarose gel containing RedSafe (1:20 000; iNtRON Biotechnology), run in Tris‐Borate‐EDTA buffer for 1 h at 80 V (Figure S10a, Supporting Information). Nucleosome assembly was performed following the same protocol described above, but using buffers without EDTA and increasing the concentrations of DNA and each histone up to 100 ng µL^−1^ and 7.5 µm, respectively, in order to properly visualize nucleosomes in a gel. After up to 1‐h incubation with 8 µm SET/TAF‐Iβ at room temperature, digestion was conducted. Samples were supplemented with 5 mm CaCl_2_ and treated with 75 U mL^−1^ of MNase (ThermoFisher) for 30 s at 37 °C. Digest was quenched by adding stop buffer 3× (60 mm EDTA, 3% SDS, pH 8) and Proteinase K (iNtRON Biotechnology) to a concentration of 0.2 mg mL^−1^, and the resulting mixture was incubated at 37 °C for 30 min. Following the reaction, DNA fragments were extracted using phenol‐chloroform and precipitated with ethanol. Purified DNA samples were supplemented with glycerol to 10% and resolved on a 1.5% agarose gel in Tris‐Borate‐EDTA buffer for 80 min at 80 V. After electrophoresis, the gel was stained with SYBR Gold (1:10 000 dilution; Invitrogen) for 30 min and imaged using the ChemiDoc system (Bio‐Rad, Figure S10b, Supporting Information).

### Circular Dichroism Spectropolarimetry

CD spectra were recorded in the far‐UV range (190–250 nm) at 25 °C on a Jasco J‐815 CD spectropolarimeter equipped with a Peltier temperature control system. 15 µm of each *X. laevis* core histone in Milli‐Q H_2_O was measured in a 1‐mm quartz cuvette. The final spectra were an average of 40 scans.

### Correlated Optical Tweezers and Confocal Fluorescence Microscopy

A commercial set‐up (LUMICKS) combining dual‐trap optical tweezers, 3‐color CFM, and microfluidics was used.^[^ ^39^ ^]^ Two DNA constructs, bacteriophage λ DNA (48,502 bp; Roche), and linearized pKYB1 vector (8370 bp; New England Biolabs) were used in this study. Both DNA constructs were end‐biotinylated and used in combination with streptavidin‐coated polystyrene beads (Spherotech) of 3.11 and 1.76 µm in diameter, with trap stiffness of ≈0.5 and ≈0.2 pN nm^−1^, respectively. 2D fluorescence images were generated by scanning the confocal volume along the area of interest and collecting the emission by single‐photon avalanche photodiodes (APDs). Pixel size was set to 100 nm and emission intensity units were given in kHz (counts•10^3^/s or photons•10^3^/s). Kymographs were constructed by collecting 1D scans along the DNA molecule over time at a constant frequency, typically between 4 and 8 Hz. Experiments were performed in low salt buffer (25 mm HEPES, 50 mm KCl, 0.1 mm EDTA, pH 7.5).

### Formation of DNA‐Histone‐Chaperone Complexes by OT and Microfluidics

A motorized stage was used to control the position of a 5‐channel microfluidic chamber, and thus rapidly change solution conditions. Individual λ‐DNA molecules were isolated using OT and stretched up to 10 pN tension (≈15.8 µm bead‐to‐bead distance) in a low salt buffer solution. While keeping the trap‐to‐trap distance constant, to avoid the formation of DNA loops, DNA molecules were incubated with a solution of 100 nm of individual histones in a low salt buffer for ≈30 s. In the case of H3, 10 mm DTT was added to avoid disulfide bond formation. Next, histone‐DNA complexes were either brought back to the buffer solution or to a solution of 5 nm chaperone in a low salt buffer to study chaperone activities. OT‐CFM experiments were performed for up to 5 min.

### Histone‐DNA Unbinding Kinetics

Histone‐DNA interaction was monitored by following the bead‐to‐bead distance, under different conditions. Bead‐to‐bead distances were determined by bright‐field imaging of the beads in combination with a bead tracking algorithm. The trap‐to‐trap distance was kept constant at all times in the absence of any feedback. The distance was chosen over force readouts because the set‐up relied on back focal plane interferometry^[^ ^40^ ^]^ to detect the forces being exerted on the beads. This made force readouts very sensitive to changes in the light path, which can be severe for different regions of the microfluidic chamber (see Figure S7a, Supporting Information).

The bead‐to‐bead distance was continuously monitored while introducing preformed histone‐DNA complexes into either buffer, chaperone solutions, or a mixture of chaperone and C*c*. The increase in distance measured over time, toward bare DNA values, was then assumed to be proportional to the amount of molecules unbinding DNA (Figure S7a, Supporting Information). Kinetic traces of 2 min were recorded for individual histone‐DNA complexes under the different conditions reported. Averaged kinetic traces were generated from at least five different single‐molecule traces and fitted to an exponential function to extract the unbinding rates (see Figure 3 and Figure S7, Supporting Information). Individual traces were manually offset in time, to remove the baseline recorded while approaching the measuring channel in the microfluidic cell (Figure S7a, Supporting Information). Traces obtained in low salt buffer were all well described by a single exponential function. However, traces recorded in chaperone solutions showed deviations from a monotonic behavior; thus, a double exponential function was used for analysis. The errors associated with the reported off‐rates represent SEM, obtained from the fits to individual single‐molecule kinetic traces.

### Force‐Extension Curves Proving Chaperone Shielding

FECs were performed on histone‐DNA complexes after 5 min incubation in either low salt buffer or chaperone solutions, where trap‐to‐trap distance was not changed to keep the ≈48 kbp DNA molecules extended, that is, ≈10 pN tension and ≈15.8 µm extension. After incubation, curves were generated by rapidly approaching one of the traps at ≈8 µm bead‐to‐bead distance (no tension applied), and then moving the trap at a constant speed (500 nm s^−1^) until reaching 50 pN of tension. Bare DNA curves were modeled by the eWLC model^[^ ^41^ ^]^

(1) $$x=L_{c}\left[1-\frac{1}{2}{\left(\frac{k_{B}T}{F\cdot L_{p}}\right)}^{1/2}+\frac{F}{S}\right]$$

With *x*, extension; *F*, force; *L*_c_, contour length; *L*_p_, persistence length; *S*, elastic modulus; and *k*_B_ *T*, Boltzmann constant times absolute temperature. The parameters that characterize the mechanical response of DNA were estimated experimentally from the eWLC fits to individual FEC of bare DNA molecules (*N* = 24) in low salt buffer, obtaining *L*_c_ = 16.45 ± 0.01 µm, *L*_p_ = 45 ± 1 nm, and *S* = 1690 ± 50 pN (mean ± SEM).

### Fluorescence Imaging and Fluorophore Brightness Estimation

Fluorescence images of fluorescently labeled chaperones were recorded at the rate of one image per min (image time ≈ 5 s) for up to 5 min, to monitor chaperone‐histone interactions while minimizing photobleaching. Images at 0 min were always taken within the first 30 s of incubation, which included the time needed to introduce the DNA‐histone construct into the microfluidic channel where imaging was performed, containing either chaperone or chaperone‐C*c* solutions. The 532 nm laser was set to ≈1 µW power at an objective and pixel time of 0.5 ms. Kymographs were constructed by collecting 1D scans along the DNA at a constant frequency, typically between 4 and 8 Hz. MFI was calculated by extracting the mean number of photons per second. In the cases where individual binding events were identified, MFI was calculated for the individual traces considering a trace thickness of four pixels, by selecting the four brightest pixels at every time point.

To estimate the count rate per fluorophore under the experimental conditions, a mixture of 10 nm H3, 10 nm H4, and 1 nm SET/TAF‐Iβ in low salt buffer was applied to the microfluidic chamber. These conditions revealed individual binding events of SET/TAF‐Iβ and DNA colocalization, which were well isolated and allowed fluorophore brightness determination. Excitation settings were kept constant, as described above. According to the labeling strategy and the dimeric nature of SET/TAF‐Iβ, the analysis reported 11 ± 3 kHz per fluorophore and 21 ± 2 kHz per dimer (Figure S4, Supporting Information).

### Nucleosome Unwrapping

Nucleosomes reconstituted on linearized pKYB1 molecules were isolated by OT. A pair of trapped beads were held in close proximity, ≈0.5 µm, under minimal flow in a solution of reconstituted nucleosomes. After 30–60 s the flow was stopped and the formation of a tether was tested by moving one bead apart from the other until reaching a force ≤1 pN at the expected extension. Typically, this procedure was repeated several times before finding a tether. Once the formation of a tether was confirmed, it was brought to a solution of 1–2 nm chaperone without applying any tension. After incubation of ≈1 min in the chaperone channel, force‐spectroscopy experiments were performed. FECs were generated by moving one of the traps at a constant speed of 20 nm s^−1^ between 1 and 32 pN. The changes in *L*_c_ were obtained by fitting the eWLC model (Equation (1)) before and after any unwrapping event while keeping *L*_p_ and *S* constant and equal to the ones obtained for bare DNA. For pKYB1, *L*_c_ = 2.818 ± 0.004 µm, *L*_p_ = 50 ± 1 nm, and *S* = 1400 ± 40 pN (mean ± SEM; *N* = 20) were estimated. Only tethers that showed the expected bead‐to‐bead distance for single tethers at 32 pN tension were used for the analysis.

Time‐correlated fluorescence imaging was used to monitor fluorescently labeled histones and chaperones during the FECs. Kymographs were performed at 4 Hz (250 ms/line scan) and with an excitation laser of 532 and 638 nm, both set to ≈1 µW power at objective. Most of the imaging time was spent without imaging, that is, of the 250 ms/line ≈200 ms were set as delay time, or waiting time, between scans. To extract the binding lifetimes of chaperones (Figure 1e,f), the advantage of having dimeric (SET/TAF‐Iβ) or pentameric (NPM) proteins was taken. Only the chaperone traces were selected, which signal (yellow) did not disappear by the end of the FEC or that did show a sudden disappearance of the signal corresponding to 2 or more fluorophores, thus minimizing the chance to analyze photobleached molecules. To identify histone eviction, events in which yellow and red signals drop simultaneously were reported, making it unlikely to be the bleaching of two fluorophores at the same time. MFI of individual fluorescence traces was obtained as described above.

### Nuclear Magnetic Resonance Measurements

1D ^1^H NMR spectra were recorded at 25 °C in a 500 MHz Bruker‐Avance III, provided with a cryoprobe, to monitor the Met80‐ε‐CH_3_ signal of reduced C*c* (13 µm), as well the base‐pairing hydrogen bond signals of a 10‐mer dsDNA (26 µm). For DNA hybridization, complementary oligonucleotide strands with the sequence 5′‐TAGCGTAACG‐3′ (Eurofins) were mixed in equimolar ratio, heated to 95 °C for 5 min, and subsequently cooled to room temperature. Samples containing both C*c* and dsDNA (1:2 ratio), in the presence or absence of the *X. laevis* histone H3 (10.4 µm), were titrated with increasing concentrations of SET/TAF‐Iβ (from 65 nm to 5.1 µm). Additional measurements with different combinations of these proteins and/or dsDNA were also performed. All measurements were taken in 3‐mm NMR tubes containing samples with a final volume of 0.2 mL. Samples were prepared in 10 mm sodium phosphate buffer (pH 7.4) supplemented with 1 mm sodium ascorbate and 1 mm tris(2‐carboxyethyl)phosphine [TCEP] to ensure the reduced state of C*c* and H3, respectively. To adjust the lock signal, 5% D_2_O was added. The water signal was suppressed according to the WATERGATE solvent suppression method. Spectra were acquired and processed using TopSpin (Bruker) and graphed using OriginPro 2018b and CorelDRAW X7.

## Conflict of Interest

The authors declare no conflict of interest.

## Author Contributions

Conceptualization: P.B., I.D.M., W.H.R.; Methodology: P.B., A.V.C., L.C.G., A.D.Q., I.D.M., W.H.R.; Investigation: P.B., A.V.C., L.C.G., A.D.Q.; Visualization: P.B., A.V.C.; Supervision: I.D.M., W.H.R.; Writing–original draft: P.B.; Writing–review & editing: P.B., A.V.C., L.C.G., A.D.Q., I.D.M., W.H.R.

## Supporting information

Supporting Information

## Data Availability

The data that support the findings of this study are available from the corresponding author upon reasonable request.
