# Supplementary material for: The Histone Chaperones SET/TAF‐1β and NPM1 Exhibit Conserved Functionality in Nucleosome Remodeling and Histone Eviction in a Cytochrome c‐Dependent Manner
Source: Adv Sci (Weinh). 2023 Aug 7;10(29):2301859. doi: 10.1002/advs.202301859 (PMC10582448; doi:10.1002/advs.202301859)
Supplement: Supplementary file 1 — Supporting Information [file ADVS-10-2301859-s001.pdf]

ADVANCED SCIENCE
Open Access

Supporting Information

for *Adv. Sci.*, DOI 10.1002/advs.202301859

The Histone Chaperones SET/TAF-1β and NPM1 Exhibit Conserved Functionality in Nucleosome Remodeling and Histone Eviction in a Cytochrome *c*-Dependent Manner

Pedro Buzón, Alejandro Velázquez-Cruz, Laura Corrales-Guerrero, Antonio Díaz-Quintana, Irene Díaz-Moreno and Wouter H. Roos**

Supporting Information

The histone chaperones SET/TAF-1β and NPM1 exhibit conserved functionality in nucleosome remodelling and histone eviction in a cytochrome *c*-dependent manner

Pedro Buzón[1,†], Alejandro Velázquez-Cruz[2], Laura Corrales-Guerrero[2], Antonio Díaz-Quintana[2], Irene Díaz-Moreno[2,] and Wouter H. Roos[1,]**

[1] Moleculaire Biofysica, Zernike Instituut, Rijksuniversiteit Groningen, Nijenborgh 4, 9747 AG Groningen, The Netherlands.

[2] Instituto de Investigaciones Químicas (IIQ), Centro de Investigaciones Científicas Isla de la Cartuja (cicCartuja), Universidad de Sevilla - Consejo Superior de Investigaciones Científicas (CSIC), Avda. Américo Vespucio 49, Sevilla 41092, Spain.

[†] Present Address: Department of Biochemistry, University of Zurich, 8057 Zurich, Switzerland

* To whom correspondence should be addressed. Email: idiazmoreno@us.es and w.h.roos@rug.nl

This file includes:

Figure S1 to S10 and Table S1

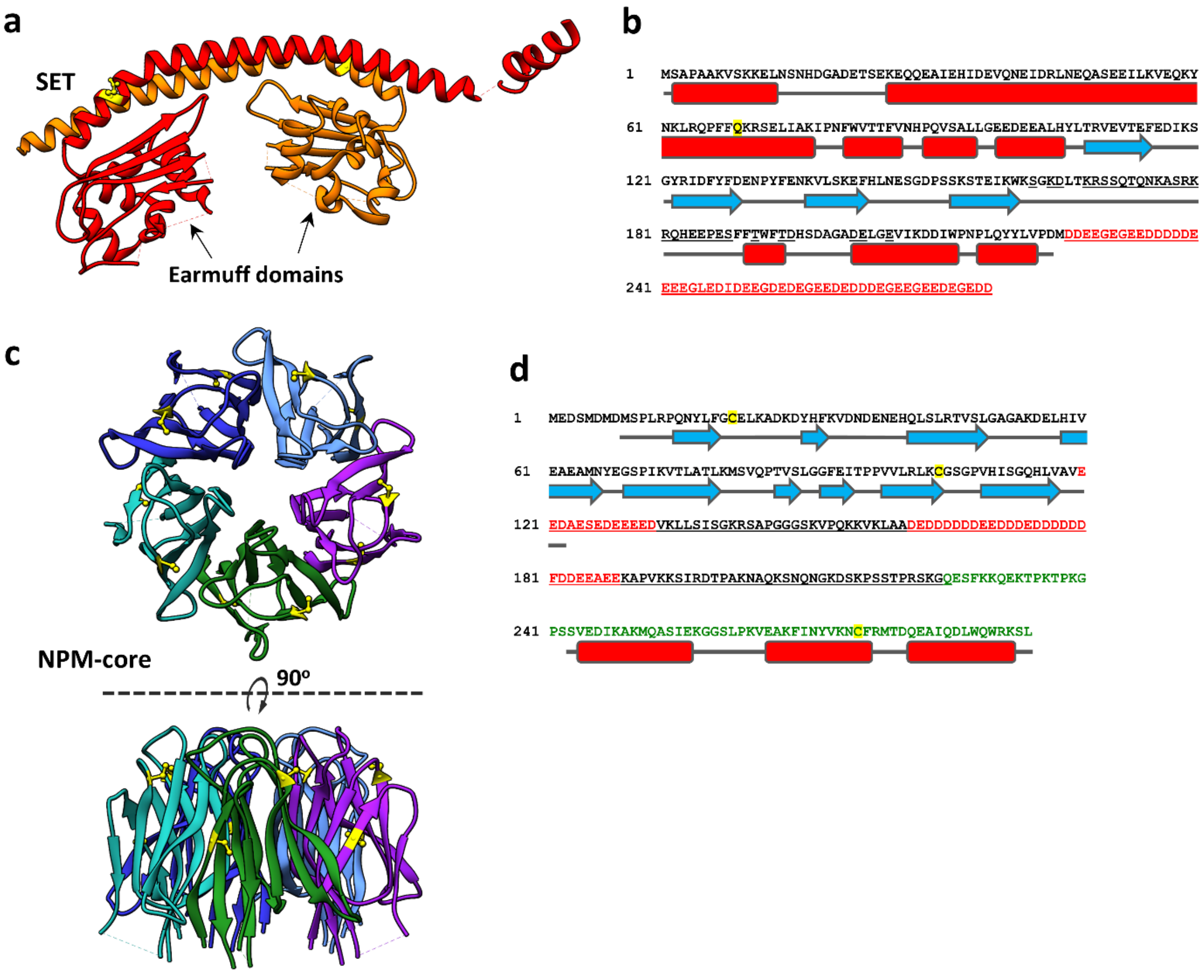

Figure S1. Structure of the histone chaperones SET/TAF-Iβ and NPM. a, Ribbon representation of SET/TAF-Iβ crystal structure with the individual monomers colored in red and orange, and the residue Q69 highlighted in yellow (disordered region is not shown). Each monomer contains an α-helix involved in the protein dimerization followed by an earmuff domain (PDB 2E50).[1] **b**, Amino acid sequence of SET/TAF-Iβ showing its secondary structure elements: α-helices (red) and β-strands (cyan). The disordered acidic region is colored in red and the residue Q69, mutated to Cys for fluorescent labeling, is highlighted in yellow. Residues involved in histone interactions are underlined. **c**, Ribbon representation of the pentameric NPM-core, amino acids sequence 1-122 (PDB 2P1B),[2] with Cys residues highlighted in yellow. View of the five-fold symmetry axis (upper panel), and side view (lower panel). **d**, Sequence of NPM showing its secondary structure elements: α-helices (red) and β-strands (cyan), according to the literature[2,3] with Cys residues highlighted in yellow. The region of the C-terminal domain identified for the interaction of NPM with DNA is colored in green.[4] The disordered acidic region is colored in red and residues involved in histone interactions are underlined. Molecular models were generated with UCSF Chimera.[5]

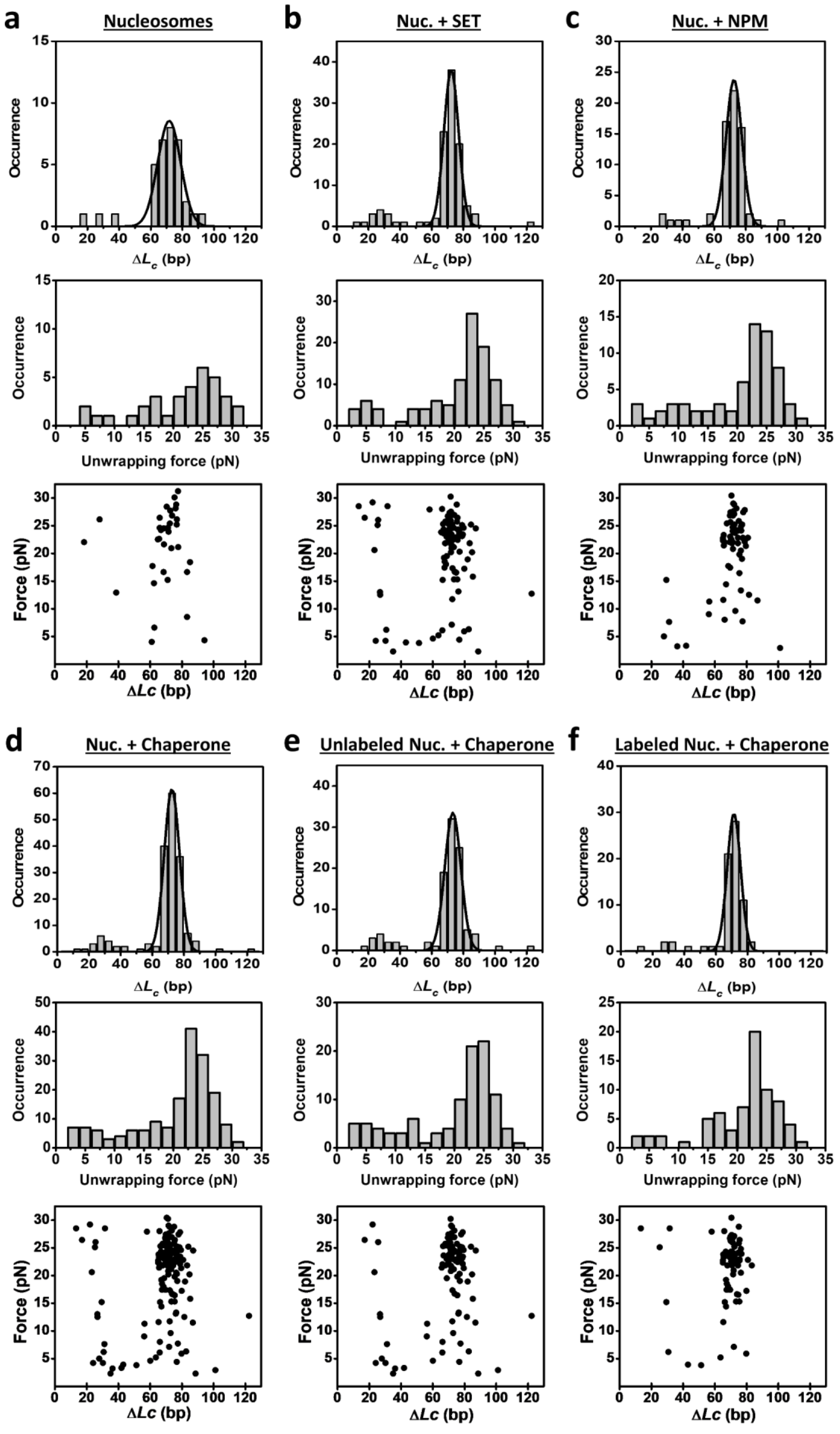

Figure S2. Nucleosome unwrapping under different conditions. Distributions of the L_c changes (top), including Gaussian fits (center ± SD), unwrapping forces (middle), and force versus ΔL_c plots (bottom) obtained from FECs performed with: **a**, only nucleosomes (N = 34; Xc = 72 ± 7 bp); **b**, nucleosomes in the presence of SET/TAF-Iβ (N = 108; Xc = 72 ± 5 bp); **c**, nucleosomes in the presence of NPM (N = 66; Xc = 72 ± 5 bp); **d**, nucleosomes in the presence of either NPM or SET/TAF-Iβ (N = 174; Xc = 72 ± 5 bp); **e**, unlabeled nucleosomes in the presence of either NPM or SET/TAF-Iβ (N = 103; Xc = 73 ± 5 bp); and **f**, fluorescently labeled nucleosomes in the presence of either NPM or SET/TAF-Iβ (N = 71; Xc = 71 ± 4 bp).

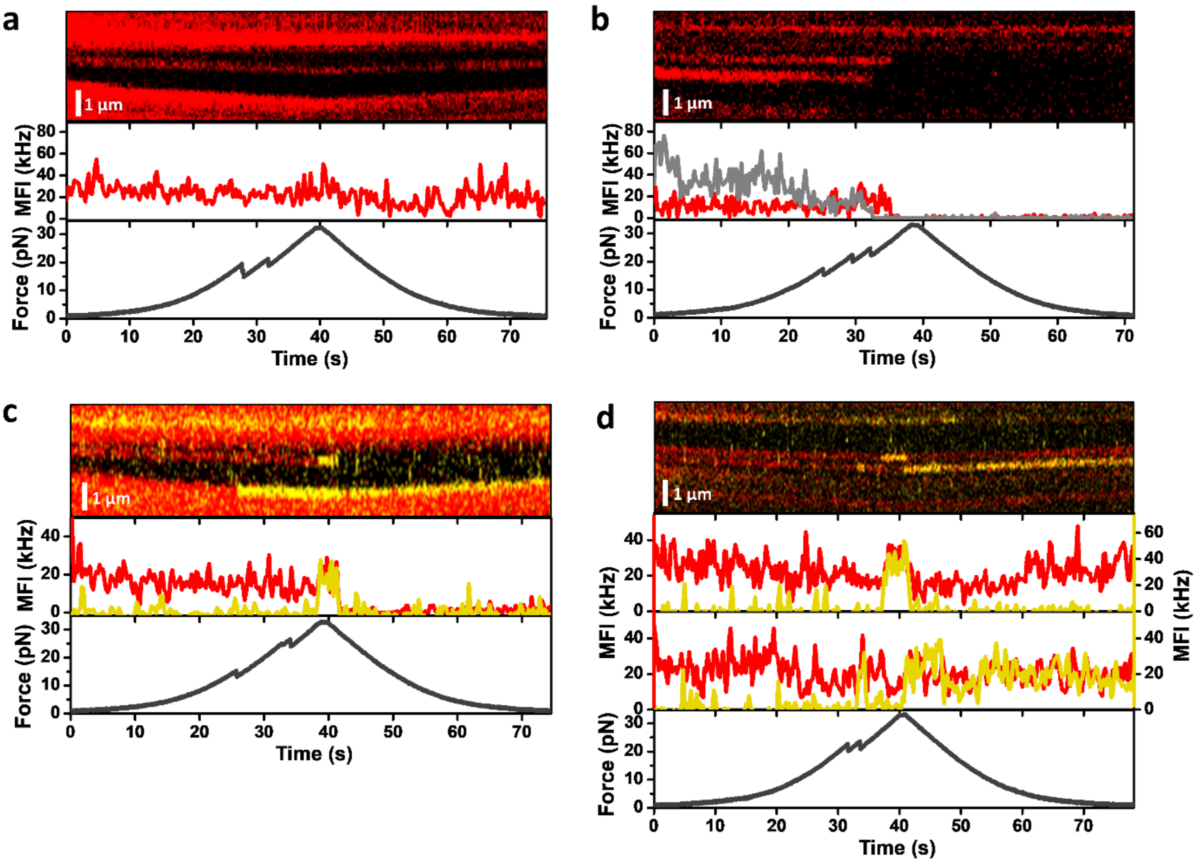

Figure S3. Correlated FEC and fluorescence imaging of fluorescently labeled histones and chaperones. a, Example of fluorescently labeled histones that remain bound after nucleosome unwrapping was detected. **b**, Example showing histone signal disappearing soon after unwrapping events were found. Middle panel shows MFI of the most upper trace (red) and lower trace (grey) of the kymograph. **c**, Example of histone (red) eviction by the chaperone SET/TAF-Iβ (yellow) in which the fluorescence signal of both histone and chaperone fall to background levels upon chaperone unbinding. **d**, Correlated FEC and fluorescence imaging showing two examples in which the histone signal (red) does not change upon unwrapping or chaperone binding (NPM; yellow). The photobleaching seen on the signal of the beads is likely to be due to the approximately 3 orders of magnitude higher intensity of the trapping laser in comparison to the excitation lasers.

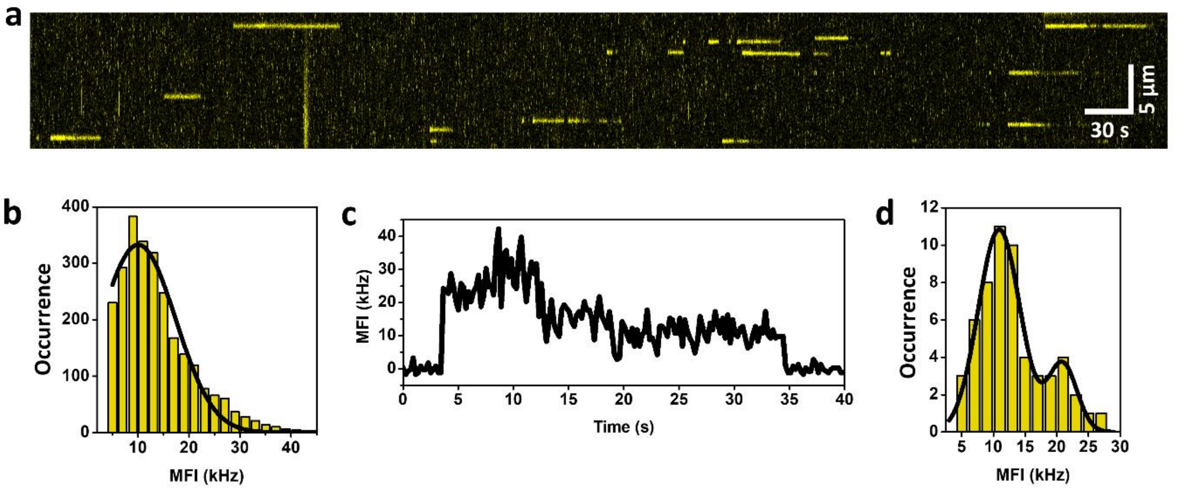

Figure S4. Fluorophore brightness. a, Kymograph recorded at 4.4 Hz (228 ms/line) in the presence of 10 nM H3, 10 nM H4, and 1 nM of fluorescently labeled SET/TAF-Iβ (Alexa Fluor 532) in low salt buffer. The kymograph shows molecules that bleach in a stepwise manner (see also panel c), as well as molecules that bind with the expected intensity of two fluorophores and then drop to background level in a single and sudden step. The latter events can be interpreted as chaperone unbinding events. **b**, Yellow bars show the MFI distribution obtained from 33 individual traces after background correction. MFI is calculated for every time point, averaging the intensity of four pixels in the distance axis, of the individual fluorescence traces. The black solid line shows the fit to a single Gaussian function. Gaussian fit reported 10 ± 7 kHz (center ± SD). **c**, Representative MFI *versus* time trace showing two discrete levels of intensity due to photobleaching; consistent with the labeling of dimeric SET/TAF-Iβ that produced the attachment of two fluorophores per dimer on average (see methods). **d**, MFI distribution constructed from the averaging of individual traces into two discrete intensity levels, high and low. Occurrence represents the total number of time-regions with constant MFI. Fit to a double Gaussian reported 11 ± 3 kHz per fluorophore and 21 ± 2 kHz per dimer (center ± SD).

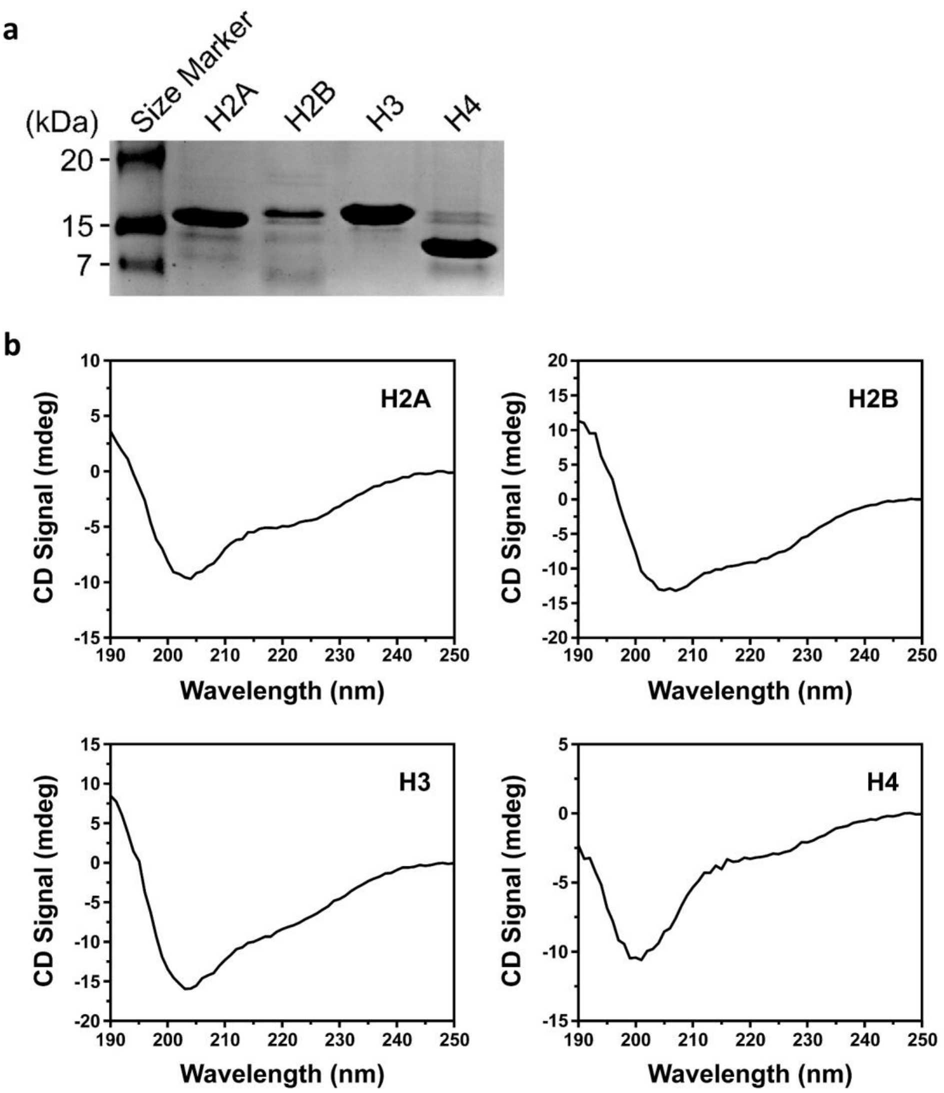

Figure S5. Purity and secondary structure analysis of histones. a, Evaluation of histone expression and purity by SDS-PAGE. Coomassie Blue-stained 15% gel loaded with the 4 core histones from *Xenopus laevis*. **b**, Far-UV CD spectra of *Xenopus laevis* core histones in Milli-Q H_2O at 15 µM concentration.

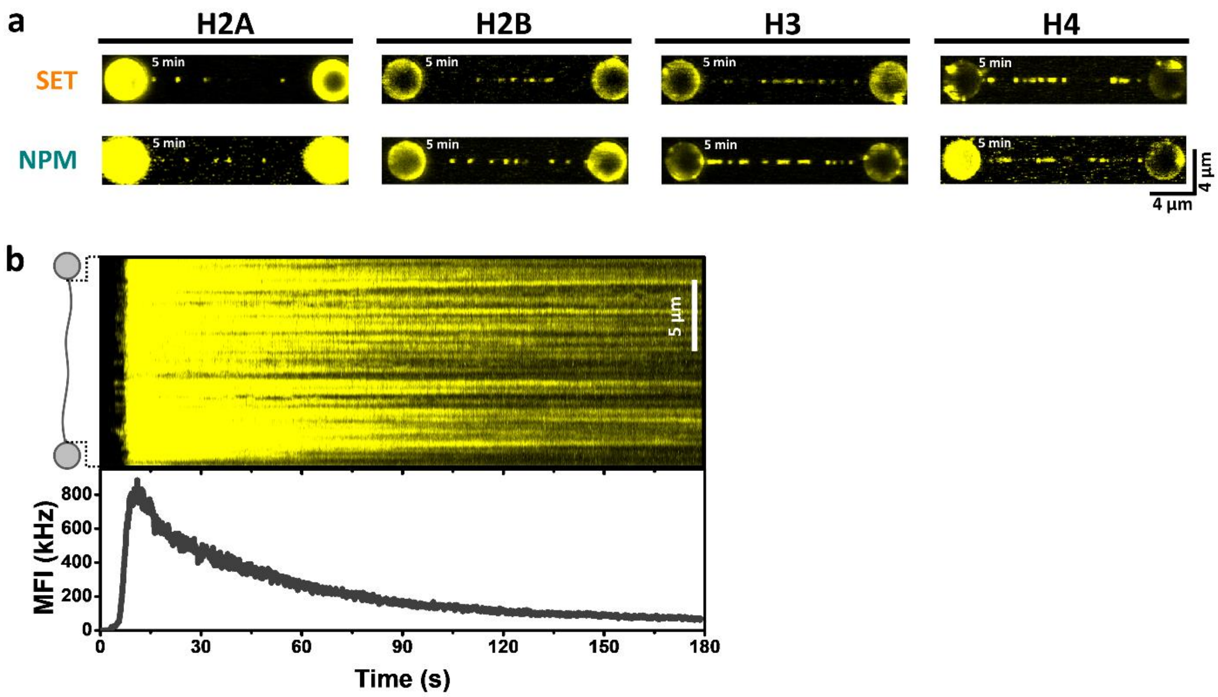

Figure S6. Visualization of histone recognition by fluorescence imaging of histone chaperones. a, Fluorescence images of 5 nM SET/TAF-Iβ (top row) and 5 nM NPM (bottom row) on DNA, after incubating the DNA-histone complex for 5 min with one type of histones (either H2A, H2B, H3, or H4, from left to right). The DNA molecules were stretched to a fixed distance, corresponding to 10 pN tension, and were then incubated in a solution of individual histones at 100 nM concentration for ~30 s. Next, histone-DNA complexes were brought to a solution of fluorescently labeled chaperone and observed for up to 5 min at the rate of one image per minute (image time ≈ 5 s). **b**, Upper panel, kymograph of a H2B-DNA complex entering the NPM solution recorded at 7.6 Hz (131 ms/line) through continuous imaging. Lower panel, mean fluorescence intensity (MFI) per line scan over time.

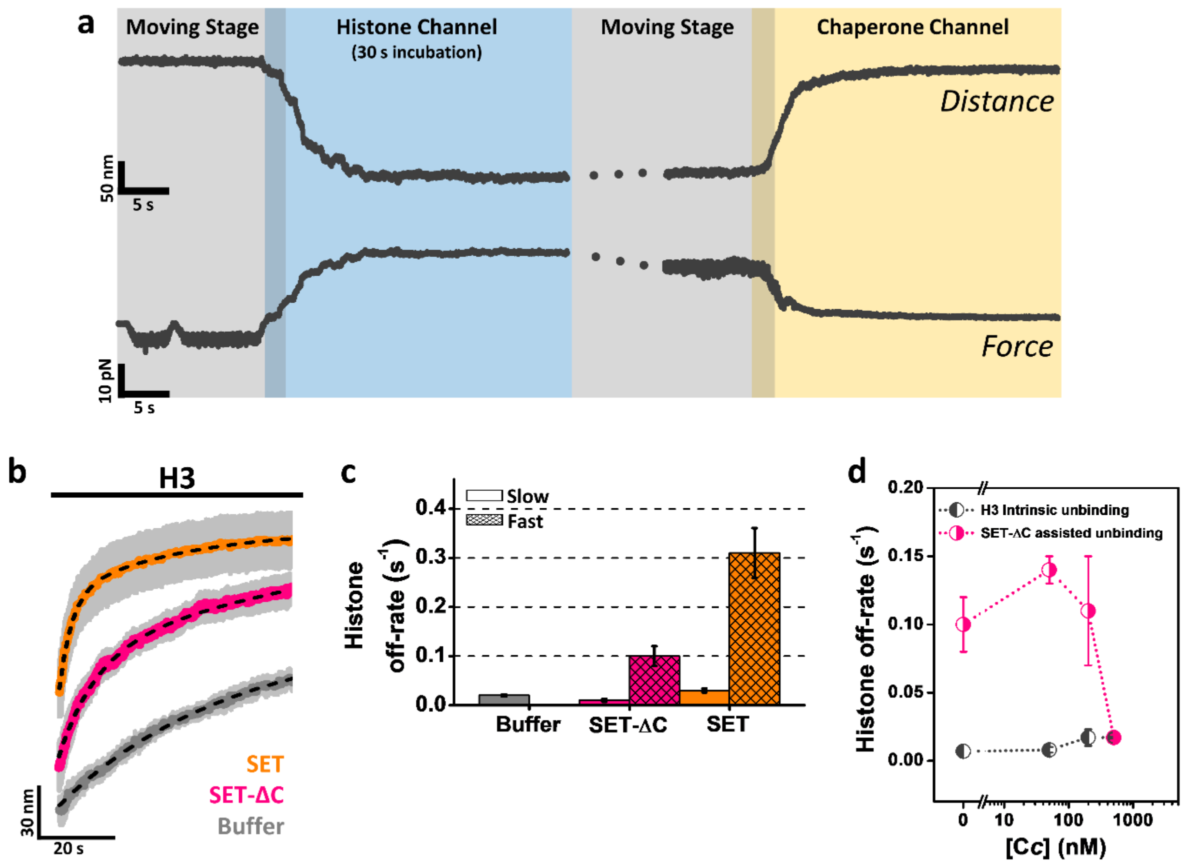

Figure S7. Binding kinetics of histone-DNA interactions mediated by histone chaperones. a, Example of a full kinetic experiment to measure histone eviction by following the changes in bead-to-bead distance (upper trace), and the corresponding force readout (lower trace). DNA molecules were stretched to a bead-to-bead distance of ~15.8 µm (~10 pN) in the buffer solution. Then, the DNA molecule is brought into a solution of individual histones at 100 nM concentration by moving the stage of the microfluidic cell holder. Incubation took place for ~30 s, where a rapid decrease in distance, DNA condensation, was observed (light blue). Next, histone-DNA complexes were brought to a solution of 5 nM chaperone (light yellow). Off-rates were calculated from 2 min traces of the bead-to-bead distance during chaperone incubation. **b**, Averaged kinetic traces of H3-DNA complexes during their incubation against buffer (grey), 5 nM SET/TAF-Iβ (orange), and 5 nM SET/TAF-Iβ-ΔC (pink). Averaged curves were generated from individual traces (N ≥ 5) and fitted to either a single or double exponential function (black dashed lines). Grey shades represent SEM. **c**, Off-rates obtained from the fits presented in (b). Error bars represent SEM, calculated from the fits to the individual kinetic traces. **d**, Off-rates at different C*c* concentrations (0, 50, 200, and 500 nM) obtained from H3-DNA complexes that were brought to a mixture solution of 5 nM SET/TAF-Iβ-ΔC and C*c*. The slow rate is depicted in dark grey, while the fast rate is colored in pink. Error bars represent SEM.

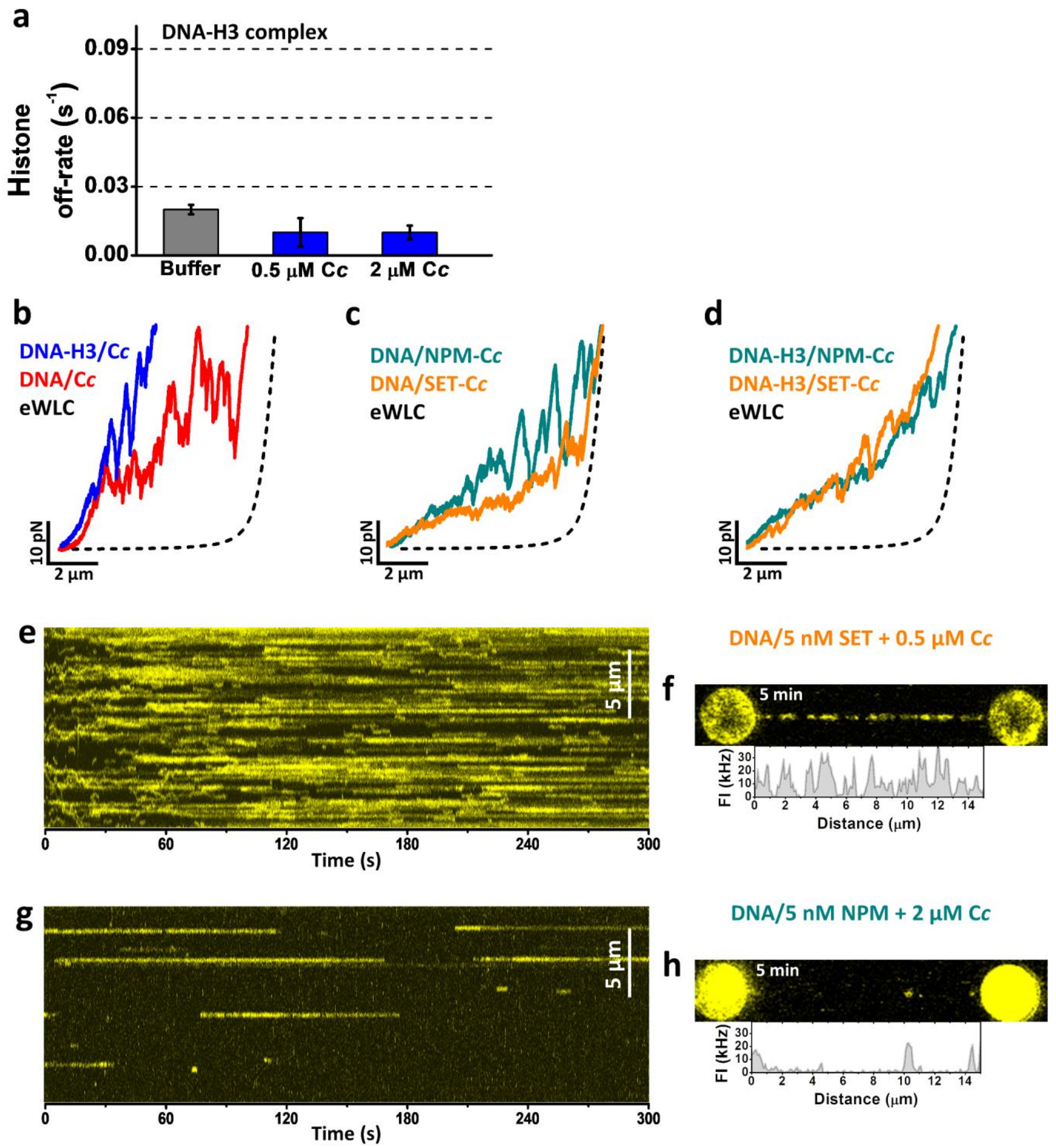

Figure S8. C*c*-DNA interactions in the presence and absence of chaperones. a, Off-rates obtained from H3-DNA complexes in a solution of either 0.5 μM or 2 μM C*c* (blue). The intrinsic unbinding rate measure for H3 is shown in grey for comparison. Error bars represent SEM, calculated from the fits of individual kinetic traces. **b**, Representative FEC of DNA and DNA-H3 in a solution of 0.5 μM C*c*, after 5 min incubation. **c**, FECs of DNA in a mixture solution of 5 nM SET/TAF-Iβ and 0.5 μM C*c* (orange), and 5 nM NPM and 2 μM C*c* (dark green), after 5 min incubation. **d**, FECs of H3-DNA complexes in the presence of 5 nM SET/TAF-Iβ and 0.5 μM C*c* (orange), and 5 nM NPM and 2 μM C*c* (dark green), after 5 min incubation. **e**, Kymograph of a DNA molecule in a solution of 5 nM SET/TAF-Iβ and 0.5 μM C*c* **f**, Fluorescence image after 5 min incubation under the same conditions described in (e). **g**, Kymograph of a DNA molecule in a solution of 5 nM NPM and 2 μM C*c*. **h**, Fluorescence image after 5 min incubation under the same conditions described in (g).

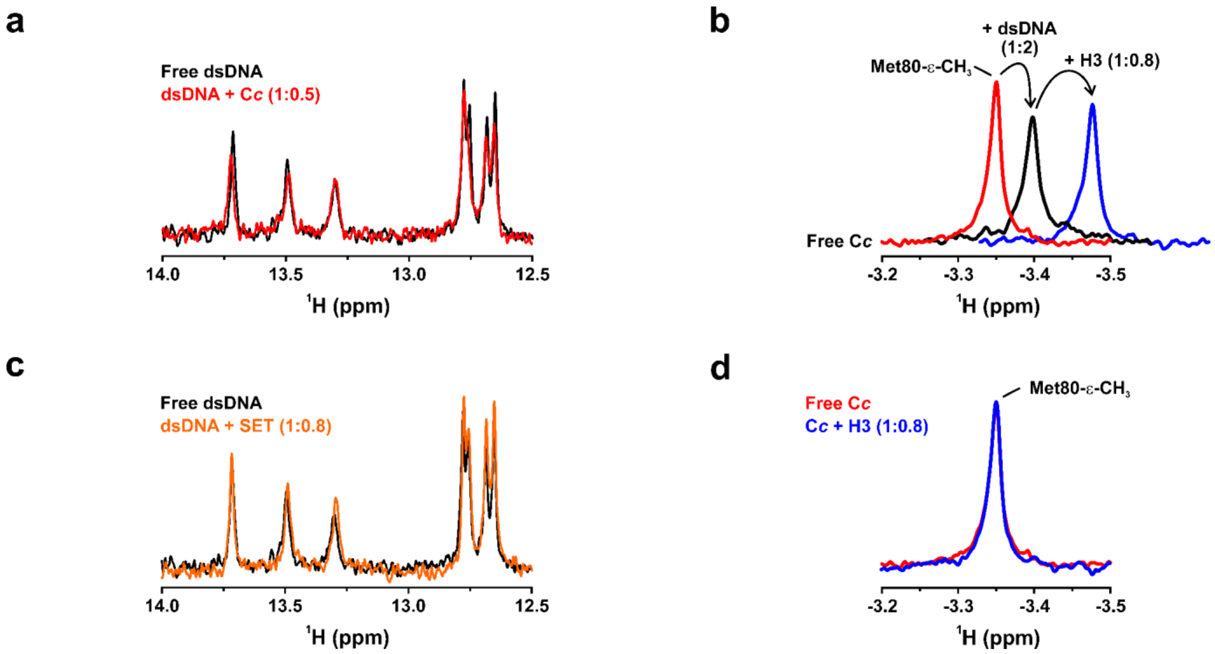

Figure S9. 1D ^{1}H NMR measurements of the interactions between DNA, histones, SET/TAF-Iβ and C*c*. a, Superimposition of the base-pairing hydrogen bond signals from spectra of dsDNA oligo, both free (black) and in combination with C*c* (red). **b,** Detailed view of spectra monitoring the Met80-ε-CH_3 signal of reduced C*c*, either free (red) or upon sequential addition of dsDNA (black) and H3 (blue). **c,** Superimposition of the base-pairing hydrogen bond signals from spectra of dsDNA oligo, both free (black) and in combination with SET/TAF-Iβ (orange). **d,** Superimposition of the Met80-ε-CH_3 signal from spectra of reduced C*c*, both free (red) and in combination with H3 (blue).

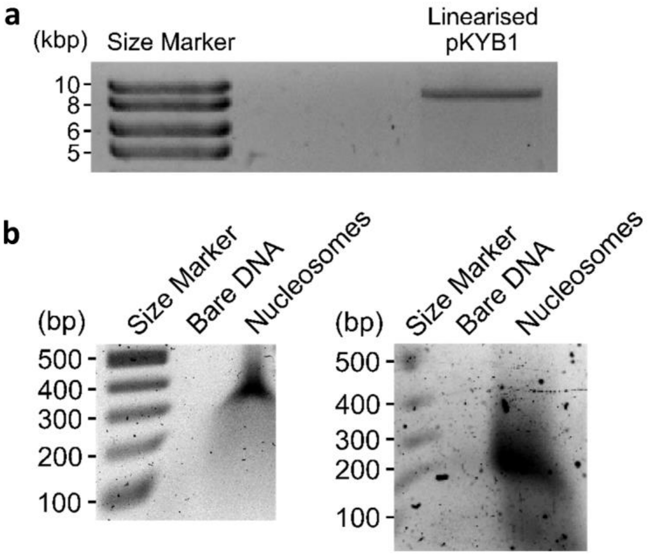

Figure S10. Large scale *in vitro* reconstitution of nucleosomes. a, Agarose gel electrophoresis of linearized pKYB1 plasmid. The DNA fragment resulting from double digestion with EcoRI and KpnI has the expected size according to the theoretical molecular weight of the plasmid (8.4 kbp). **b,** Micrococcal nuclease digestion of reconstituted nucleosomes. The MNase assay was performed by using linearised pKYB1 plasmid either alone or upon nucleosome assembly with recombinant *Xenopus laevis* core histones and SET/TAF-Iβ. Two independent experiments are shown where dinucleosomes (left) or mononucleosomes (right) were observed.

Table S1. Nucleosome (Nuc.) unwrapping events identified under various experimental conditions.

Experimental conditions	N unwrapping events at high force (>10 pN)	N unwrapping events at low force (<10 pN)[#]	% unwrapping events at low force (<10 pN)[§]
**Nuc. Only**	**30**	**4**	**13**
**Nuc. + SET**	**94**	**14**	**15**
**Nuc. + NPM**	**57**	**9**	**16**
**Nuc. + chaperone**	**151**	**23**	**15**
**Unlabeled Nuc. + chaperone**	**86**	**17**	**20**
**Labeled Nuc. + chaperone**	**65**	**6**	**9**

[#] Number of experiments where next to high force unwrapping events (column to the left) also low force unwrapping events were recorded. [§] Percentage of experiments where high force unwrapping events (> 10 pN) were preceded by low force unwrapping events (<10 pN).

References

[1] S. Muto, M. Senda, Y. Akai, L. Sato, T. Suzuki, R. Nagai, T. Senda, M. Horikoshi, Relationship between the structure of SET/TAF-Iβ/INHAT and its histone chaperone activity. *Proceedings of the National Academy of Sciences* **104**, 4285 (2007).

[2] H. H. Lee, H. S. Kim, J. Y. Kang, B. I. Lee, J. Y. Ha, H. J. Yoon, S. O. Lim, G. Jung, S. W. Suh, Crystal structure of human nucleophosmin-core reveals plasticity of the pentamer–pentamer interface. *Proteins: Structure, Function, and Bioinformatics* **69**, 672-678 (2007).

[3] C. G. Grummitt, F. M. Townsley, C. M. Johnson, A. J. Warren, M. Bycroft, Structural Consequences of Nucleophosmin Mutations in Acute Myeloid Leukemia. *Journal of Biological Chemistry* **283**, 23326-23332 (2008).

[4] L. Federici, A. Arcovito, G. L. Scaglione, F. Scaloni, C. Lo Sterzo, A. Di Matteo, B. Falini, B. Giardina, M. Brunori, Nucleophosmin C-terminal leukemia-associated domain interacts with G-rich quadruplex forming DNA. *Journal of Biological Chemistry* **285**, 37138-37149 (2010).

[5] E. F. Pettersen, T. D. Goddard, C. C. Huang, G. S. Couch, D. M. Greenblatt, E. C. Meng, T. E. Ferrin, UCSF Chimera--a visualization system for exploratory research and analysis. *J Comput Chem* **25**, 1605-1612 (2004).
